# Acetylcholinesterase overexpression mediated by oncolytic adenovirus exhibited potent anti-tumor effect

**DOI:** 10.1186/1471-2407-14-668

**Published:** 2014-09-15

**Authors:** Haineng Xu, Zhengxuan Shen, Jing Xiao, Yu Yang, Weidan Huang, Zhiming Zhou, Jiani Shen, Yizhun Zhu, Xin-Yuan Liu, Liang Chu

**Affiliations:** State Key Laboratory of Cell Biology, Institute of Biochemistry and Cell Biology, Shanghai Institutes for Biological Sciences, Chinese Academy of Sciences, Shanghai, 200031 China; Department of Pharmacology, School of Pharmacy, Fudan University, Shanghai, 201203 China; College of Life Science, Henan Normal University, Xinxiang, Henan 453007 China; Xinyuan Institute of Medicine and Biotechnology, Zhejiang Sci-Tech University, Hangzhou, 310018 China

**Keywords:** AChE, Oncolytic adenovirus, Gastric cancer, Apoptosis

## Abstract

**Background:**

Acetylcholinesterase (AChE) mainly functions as an efficient terminator for acetylcholine signaling transmission. Here, we reported the effect of AChE on gastric cancer therapy.

**Methods:**

The expression of AChE in gastric cancerous tissues and adjacent non-cancerous tissues was examined by immunohistochemistry. Gastric cancer cells were treated with AChE delivered by replication-deficient adenoviral vector (Ad.AChE) or oncolytic adenoviral vector (ZD55-AChE), respectively, followed by measurement of cell viability and apoptosis by MTT assay and apoptosis detection assays. In vivo, the tumor growth of gastric cancer xenografts in mice treated with Ad.AChE or ZD55-AChE (1 × 10^9^ PFU) were measured. In addition, the cell viability of gastric cancer stem cells treated with Ad.AChE or ZD55-AChE were evaluated by MTT assay.

**Results:**

A positive correlation was found between higher level of AChE expression in gastric cancer patient samples and longer survival time of the patients. Ad.AChE and ZD55-AChE inhibited gastric cancer cell growth, and low dose of ZD55-AChE induced mitochondrial pathway of apoptosis in cells. ZD55-AChE repressed tumor growth in vivo, and the anti-tumor efficacy is greater than Ad.AChE. Moreover, ZD55-AChE suppressed the growth of gastric cancer stem cells.

**Conclusion:**

ZD55-AChE represented potential therapeutic effect for human gastric cancer.

**Electronic supplementary material:**

The online version of this article (doi:10.1186/1471-2407-14-668) contains supplementary material, which is available to authorized users.

## Background

Acetylcholinesterase (AChE) terminates cholinergic signaling transmission by hydrolyzing the signaling molecule acetylcholine (ACh), which acts as a stimulus signaling transmitter in neuronal and non-neuronal cholinergic systems. It binds to nicotinic or muscarinic acetylcholine receptors (nAChR or mAChR), and promotes Ca^2+^ entry into neuronal and non-neuronal cells, thus stimulating their biological activities [1]. Besides its canonical functions, ACh can also over-activate the nicotinic acetylcholine receptor, which leads to angiogenesis, migration and proliferation of cancer cells [1, 2]. Abnormal expression of AChE protein was found in several types of cancer [3, 4]. Hepatocellular carcinoma patients with low expression of AChE exhibited poor prognosis [5]. Recently, AChE was reported to be involved in apoptosis [6]. Increased intracellular AChE expression was observed in various cell lines undergoing apoptosis, as well as in animal models of diabetes and Parkinson’s disease [6–8]. Critical proliferation pathways including PI3K/Akt pathway in cancer cells were inhibited by AChE overexpression [5]. However, there is no report on cancer therapy using AChE protein.

Gastric cancer is one of the leading causes of cancer-related deaths worldwide. The prevalence of gastric cancer is related to an unhealthy diet, smoking and *Helicobacter pylori* infection [9, 10]. In 2008, about 989,600 patients were diagnosed with gastric cancer and 738,000 patients died [11]. Eastern Asia is one of the regions with a high incidence of gastric cancer. Since early stage gastric cancer may be vague and overlooked, many patients are diagnosed in later life, and the conventional treatments are ineffective for those cases.

In the present study, we detected the expression of AChE in gastric cancerous tissues (CT) and the adjacent non-cancerous tissues (ANCT). Gastric cancer samples presented a low AChE expression compared to the non-cancerous samples, and patients with higher AChE levels showed a longer survival. Overexpression of AChE by an oncolytic adenoviral vector (ZD55-AChE) significantly inhibited gastric cancer cell proliferation and reduced growth of gastric tumors in mice. In addition, ZD55-AChE suppressed gastric cancer stem cell growth. Our work demonstrated for the first time that AChE gene mediated by an oncolytic adenovirus is effective for suppressing digestive system cancers.

## Methods

### Patient samples

Ninety-six gastric and seven respective adjacent non-cancerous tissues were obtained anonymously from Zhongshan Hospital (Fudan University, Shanghai, China) and Xinhua hospital (Jiaotong Universtiy, Shanghai, China). All the human samples were obtained with informed consent and approval for usage was received from the ethics committee of Zhongshan Hospital and Xinhua Hospital. Studies upon these samples were approved and handled in accordance with the Institutional Review Board of Institute of Biochemistry and Cell Biochemistry, Shanghai Institutes for Biological Sciences, Chinese Academy of Sciences.

### Immunohistochemistry

Patient samples or tumor xenografts were processed into tissue chips by Shanghai Superchip Biotechnology Corporation (Shanghai, China) and subjected to immunohistochemistry assay according to the standard protocol [12]. Level of AChE expression was set as values range from 0 to 3 according to the intensity, and expression of AChE were calculated as (labelling intensity × percentage of positive cells). Antibody (clone AE1, lot number MAB303) to AChE was purchased from Millipore (Billerica, MA).

### Cell culture and primary fibroblast culture

Gastric cancer NCI-N87, MGC80-3 cells, liver cancer Huh-7, SMMC-7721 cells, cervical cancer HeLa cells, colon cancer SW480 cells, pancreatic cancer BxPc-3 cells, embryo kidney HEK293 and 293 T cells were grown in Dulbecco’s modified Eagle’s medium (DMEM, Gibico, Grand Island, NY) containing 10% fetal bovine serum (FBS, Gibico) and 1× penicillin-streptomycin (Sigma-Aldrich, St. Louis, MO). Gastric cancer AGS cells and the normal gastric epithelial cell line GES-1 cells were grown in Roswell Park Memorial Institute (RPMI) 1640 medium (Gibico) containing 10% FBS and 1× penicillin-streptomycin. Colon cancer HCT116 cells were grown in McCoy’s 5A medium modified (Gibico) containing 10% FBS and 1× penicillin-streptomycin. Among these cell lines, Huh-7 and HEK293 cells were obtained from RIKEN Cell Bank (Tsukuba, Japan) and ATCC (Manassas, Virginia, U.S.), respectively. GES-1 cells were a gift from Xijing digestive disease hospital laboratory of The Fourth Military Medical University (Xi’an, China) and all the other cells were purchased from Cell Bank of the Chinese Academy of Sciences (Shanghai, China).

For primary fibroblast culture, the gastric cancer tissues were washed with Hank’s solution (Beyotime, Nantong, China), cut into small pieces and plated into polylysine coated flask. The culture medium is DMEM supplemented with 20% FBS, 100 U/L penicillin-streptomycin, 5 μg/mL amphotericin B (Sigma-Aldrich), 5 μg/mL insulin (Sigma-Aldrich), 50 ng/mL epidermal growth factor (EGF, peprotech, Rocky Hill, NJ) and 50 ng/mL basic fibroblast growth factor (bFGF, peprotech). The fibroblast cells were trypsinized around a month later and moved into a new flask for amplification.

### Adenovirus construction

AChE gene was amplified by PCR assay, using AChE forward primer 5’-ATCGAAGCTTATGAGGCCCCCGCAGT-3’ and reverse primer 5’-GTACTCTAGATCACAGGTCTGAGCAGC-3’, with pGS-AChE (T form of AChE) as the template (a gift from Dr. Oksana Lockridge, Eppley Institute, University of Nebraska Medical Center). The PCR fragments were restricted by *HindIII* and *XbaI* and inserted into pCA13 shuttle plasmid to form pCA13-AChE. The CMV-AChE-SV40 cassette in pCA13-AChE was restricted by *BglII* and inserted into pZD55 shuttle plasmid to generate pZD55-AChE. pCA13, pCA13-AChE, pZD55 and pZD55-AChE were co-transfected into HEK293 cells with pBGHE3, respectively. Viral plaques were picked, amplified and purified by gradient CsCl solution centrifugation. The virus titer was measured using Virus titer detection kit (Vector Gene Biotechnology, Beijing, China). The genome of the adenovirus was obtained by using Cell-blood genome extraction kit (Generay, Shanghai, China). AChE gene was identified by PCR assay using the AChE primers mentioned above.

### Adenovirus infection

For AChE determination assay, cells were infected with Ad.vector, Ad.AChE, ZD55 or ZD55-AChE at the indicated MOIs. 48 hours post-infection, cell lysis and medium were collected and subjected to AChE determination.

For cell viability assay, cells were infected with Ad.vector, Ad.AChE, ZD55 or ZD55-AChE at different MOIs for 96 hours and then subjected to cell viability detection.

### AChE determination

Measurement of AChE activity was performed according to a modified Ellman’s assay [13]. Briefly, 1×10^6^ cells were washed with cold PBS, incubated with 50 μL lysis buffer (1 M NaCl with 0.5% Tween, 0.05 M K_2_HPO_4_, 0.01 M KH_2_PO_4_) for 30 min, and then dissociated by ultra-sonication. 20 μL cell lysate or culture medium was incubated with 300 μL 0.1 M PB buffer (pH 8.0), 10 μL 0.01 M DTNB and 1.5 μL 0.01 M iso-OMPA for 30 min at room temperature. 20 μL 0.075 M acetylthiocholine was added into the mixture and the absorbance at wavelength 405 nm was measured instantly and then every 5 minutes for a total of 6 times. Experiments were repeated 3 times.

### Acetylcholine production level determination

Measurement of human intracellular ACh levels were performed using an ACh determination ELISA Kit (HEA004, Bogoo, shanghai, China) according to the manufacturer’s protocol. Cells were collected at a concentration of 10^6^ cell/mL and washed by PBS for three times. After repeated freezing and thawing, cells were lysed and the supernatants were applied for measurement in 96-well plates pre-coated with an ACh monoclonal antibody. ACh standard solutions with diluted concentrations were used for standard curve. The results were reflected by absorbance value at a wavelength of 450 nm. Experiments were repeated 3 times.

### Cell viability detection

Cells were incubated with 10 μL 3-(4, 5-dimethylthiazol-2-yl)-2, 5-diphenyl tetrazolium bromide (MTT, 4 mg/mL, Beyotime) for 4 hours. After the removal of supernatant, the precipitate was dissolved in 100 μL DMSO, and the absorbance at the wavelength of 595 nm and 630 nm was measured. The cell viability was calculated as OD_595_-OD_630_. Experiments were repeated 3 times.

### Western blot

Total cellular proteins were denatured at 100°C for 10 min, separated by electrophoresis, transferred onto polyvinylidene fluoride (PVDF) membrane (Millipore, Billerica, MA) and incubated with antibodies. Antibodies to AChE (sc-11409), procaspase 3 (sc-69456) and PARP (sc-7150) were obtained from Santa Cruz Biotechnology (Santa Cruz, CA), antibodies to procaspase 8 (#9746) and procaspase 9 (#9508) were obtained from Cell Signaling Technology (Beverly, MA), and antibody to GAPDH (CW002A) was obtained from CoWin Bioscience (Beijing, China). Blots were incubated with horseradish peroxidase-conjugated secondary antibodies (Santa Cruz Biotechnology) and visualized by enhanced chemiluminescence (Pierce, Rockford, IL). Experiments were repeated 3 times.

### Hoechst 33258 staining, cell cycle detection and Annexin V/PI staining assay

For Hoechst 333258 staining, cells were fixed with 4% paraformaldehyde (PFA) for 15 min, washed with PBS, and incubated with 1 μg/mL Hoechst33258 (Molecular Probe, Grand Island, NY) for 1 min in the dark.

To detect the cell cycle distributions, cells were trypsinized and fixed with 70% ethanol at -20°C overnight. The fixed cells were washed with PBS twice, digested with 20 μg/mL RNase A (Sigma-Aldrich) at 37°C, and incubated with 50 μg/mL propidium iodide (PI, Sigma-Aldrich) for 30 min in the dark.

Apoptotic cells were analyzed using an Annexin V-FITC/PI kit (Biovision, Milpitas, CA) according to the manufacturer’s protocol. Cells were trypsinized, washed with binding buffer twice, incubated with 5 μL Annexin V and 10 μL PI for 15 min in the dark, and subjected to flow cytometry analysis.

### Mitochondrial membrane potential alteration detection

Mitochondrial membrane potential alteration was detected using a JC-1 staining kit (Beyotime) according to the manufacturer’s protocol. Cells were trypsinized, resuspended in 500 μL corresponding culture medium, then mixed with 500 μL JC-1 solution, incubated at 37°C in the dark for 30 min, and analyzed with flow cytometry.

### Cell sorting

AGS cells were collected and stained with APC conjugated CD44 antibody (Miltenyi, Bergisch Gladbach, Germany) for 10 min at 4°C in the dark. Cells needed to be continuously shaken during the period of antibody incubation to avoid precipitation. After that, cells were washed and sorted. AGS cells stained with APC conjugated mouse isotype antibody (Miltenyi) or without staining were used as negative controls.

### Animal experiments

All the experiments were approved by the Institutional Animal Care and Use Committee of Institute of Biochemistry and Cell Biochemistry, Shanghai Institutes for Biological Sciences, Chinese Academy of Sciences, and manipulated according to the U.S. Public Health Service Policy on Humane Care and the Use of Laboratory Animals.

AGS cells failed to form subcutaneous tumors at 1 × 10^7^ cells/mouse. We then mixed AGS cells with matrigel (BD Bioscience, San Jose, CA) at a ratio of 1:1 to enhance the tumor formation ability of the cells. Unfortunately, AGS cells were still unable to form tumors. The experiments to establish AGS xenograft tumors were performed four times without success.

Gastric cancer cell line MGC80-3 cells were utilized for establishing subcutaneous xenograft tumors. Four-week old female nude mice were subcutaneously injected with MGC80-3 cells at 4 × 10^6^ cells/mouse. When the size of tumors reached around 90 mm^3^, mice were divided into 5 groups by R software. The mice were intratumorally injected with saline or indicated adenoviruses (1 × 10^9^ PFU) every other day. The size of tumors was measured every three days after the treatment, and the volume was calculated as length × width × width/2.

### Statistical analysis

Overall survival rate of gastric cancer patients was calculated with Kaplan-Meier. All the statistical data were expressed as mean ± SD. Statistical analysis between two groups were performed with students’ *t* test in R software. P < 0.05 was considered as significant difference.

## Results

### The expression of AChE is low in gastric cancer patient samples and is associated with survival in patients

**Figure 1:**
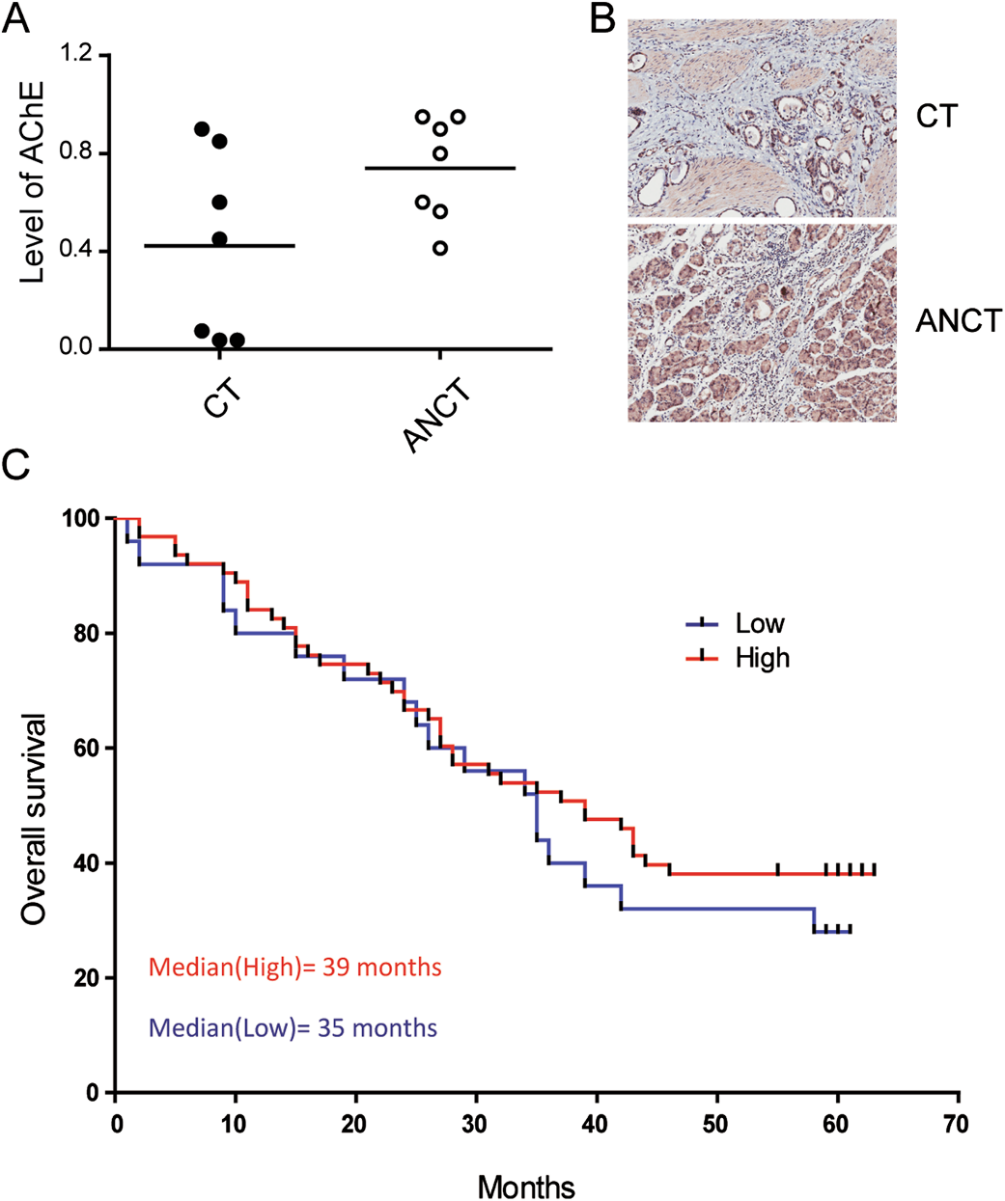
**The AChE expression in gastric cancers. (A)** Expression of AChE in 7 gastric cancerous tissues (CT) and adjacent non-cancerous tissues (ANCT) as measured by immunohistochemistry staining. The expression of AChE was calculated as labelling intensity × percentage of positive cells and is displayed in a scatter plot. **(B)** Representative immunohistochemistry staining images of AChE expression in gastric samples (100×). **(C)** Overall survival rate of gastric cancer patients correlates with the expression level of AChE protein. n = 89.

To detect the expression of AChE in gastric cancerous tissues (CT) and the adjacent non-cancerous tissues (ANCT), we performed immunohistochemistry staining assay in paired tissues. Relatively lower expression level of AChE was observed in gastric cancer tissues compared to the adjacent non-cancerous tissues (Figure 1A, B). In addition, gastric cancer patients with higher AChE expression levels exhibited longer survival time (Figure 1C).

### Replication-deficient adenovirus carrying AChE gene inhibited cancer cell growth

**Figure 2:**
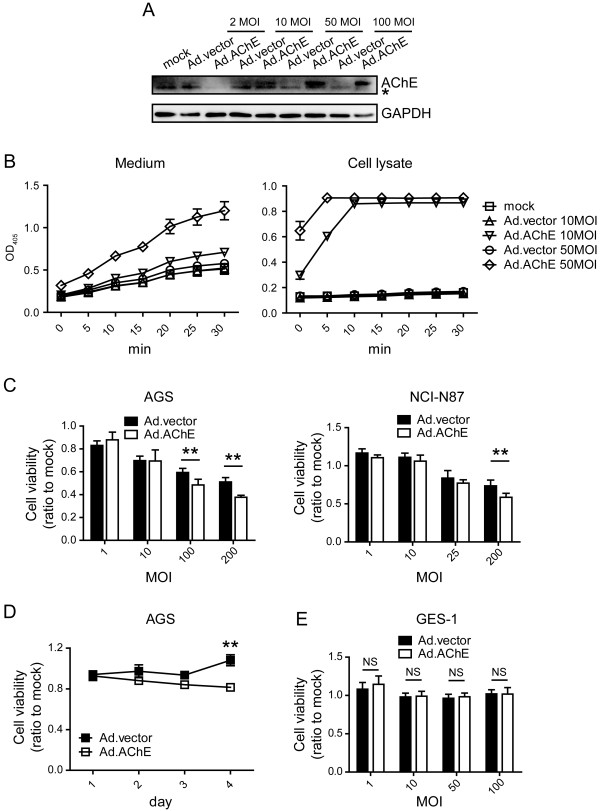
**Replication-deficient adenovirus expressing AChE inhibited gastric cancer cell growth. (A)** Western blot analysis of AChE expression in Ad.AChE infected AGS cells. GAPDH was used as an internal control. *, non-specific band. **(B)** 48 hours after AGS cells were infected with Ad.AChE, AChE activity in the medium (left) and cell lysates (right) were detected at the indicated time points. Data are presented as mean ± SD (n = 3). **(C)** Cell viability of AGS and NCI-N87 cells 4 days after the indicated MOI of adenovirus infection measured by MTT assay. **(D)** Ad.AChE suppressed AGS cell growth at a MOI of 100 as measured by MTT assay. **(E)** Detection of the cytotoxicity of Ad.AChE on GES-1 cells by MTT assay. MTT experiments were repeated 3 times. Data in **C**-**E** are shown as fold change relative to that of mock-treated cells. All data shown represent mean ± SD (n = 3). **P < 0.01, NS: non-significant.

To determine whether overexpression of AChE could inhibit cell growth, the replication-deficient adenovirus carrying AChE gene (Ad.AChE) was constructed (Additional file 1: Figure S1A, B). Activated AChE protein was expressed in Ad.AChE-infected gastric cancer cell line AGS cells, and can be secreted to culture medium (Figure 2A, B). The AGS cell growth and another gastric cancer cell line NCI-N87 cell growth were significantly inhibited by Ad.AChE at a high MOI level (100 or 200) (Figure 2C, D). Ad.AChE also decreased cell viability in other digestive system cancers, including liver cancer cell line (Huh-7 and SMMC-7721), colon cancer cell line (SW480) and pancreatic cancer cell line (BxPc-3) (Additional file 1: Figure S1C, D). Ad.AChE did not show cytotoxicity to normal gastric epithelial GES-1 cells or primary fibroblasts (Figure 2E and Additional file 1: Figure S1E, F). Notably, Ad.AChE was not able to inhibit most cancer cell growth at low MOI level, such as MOI of 1 or 10, compared to the control adenovirus (Ad.vector). In addition, endogenous level of ACh in various cell lines were detected. ACh production level of AGS cells appeared to be the highest one among the gastric epithelia cells (Additional file 2: Figure S2A) and that of pancreatic BxPC-3 cells is relatively high (Additional file 2: Figure S2B).

### AChE expressed by oncolytic adenoviral vector presented potential anti-tumor efficiency in vitro and in vivo

**Figure 3:**
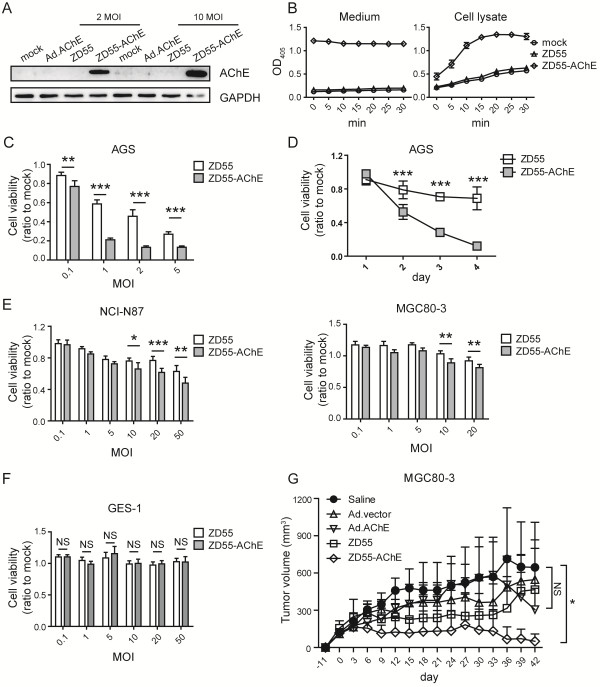
**AChE overexpression mediated by oncolytic adenovirus ZD55 inhibited cancer cell growth. (A)** Western blot detection of AChE expression in AGS cells infected with adenoviruses for 48 hours. GAPDH was used as an internal control. **(B)** 48 hours after AGS cells were infected with ZD55-AChE, AChE activity in the medium (left) and cell lysates (right) were detected at the indicated time points. Data are presented as mean ± SD (n = 3). **(C)** Cell viability of AGS cells 4 days after the indicated MOI of ZD55-AChE infection measured by MTT assay. **(D)** ZD55-AChE suppressed AGS cell growth at a MOI of 2 as measured by MTT assay. **(E)** Cell viability of NCI-N87 and MGC80-3 cells 4 days after the indicated MOI of ZD55-AChE infection measured by MTT assay. **(F)** Detection of the cytotoxicity of ZD55-AChE on GES-1 cells 4 days after infection by MTT assay. **(G)** Tumor growth curve of the adenoviruses-treated subcutaneous MGC80-3 xenografts. Day 0, animals were intratumorally injected with saline or adenoviruses. Data are expressed as mean ± SD (n = 6). MTT experiments were repeated 3 times. Data in **C**-**F** are shown as fold change relative to that of mock-treated cells. All data shown represent mean ± SD (n = 3). *P < 0.05, **P < 0.01, ***P < 0.001, NS: non-significant.

Oncolytic adenoviral vector was widely used in cancer therapy with good antitumor effect. To detect the potential cancer therapeutic function of AChE, we constructed an oncolytic adenovirus carrying AChE gene (ZD55-AChE) (Additional file 3: Figure S3A, B). Overexpression of activated AChE protein in AGS cells significantly decreased cell viability as measured by MTT assay (Figure 3A-D). ZD55-AChE suppressed cell growth in gastric cancer NCI-N87 and MGC80-3 cells at a MOI of 10 (Figure 3E). We also detected the function of ZD55-AChE in the pancreatic cancer cell line BxPc-3, liver cancer cell line Huh-7 and SMMC-7721, and colon cancer cell line SW480 and HCT116, and found that ZD55-AChE inhibited BxPc-3, Huh-7 and HCT116 cell growth at a MOI of 1. However, ZD55-AChE cannot significantly suppress SW480 cell growth at the low MOI level (Additional file 3: Figure S3C). Importantly, ZD55-AChE did not affect normal gastric epithelial GES-1 cells or primary fibroblast cell proliferation (Figure 3F and Additional file 3: Figure S3D, E).

Next, we sought to explore the anti-tumor effect of ZD55-AChE in vivo. Since AGS cells failed to form subcutaneous xenografts, we evaluated the anti-tumor effect of AChE in the MGC80-3 xenografts. Ad.AChE, ZD55-AChE and the control viruses were intratumoral injected, respectively. The tumor growth was significantly reduced by ZD55-AChE, as compared with the control animals receiving saline. However, Ad.AChE did not inhibit the growth of tumors (Figure 3G and Additional file 4: Figure S4).

### ZD55-AChE induced gastric cancer cell apoptosis through mitochondrial pathway

**Figure 4:**
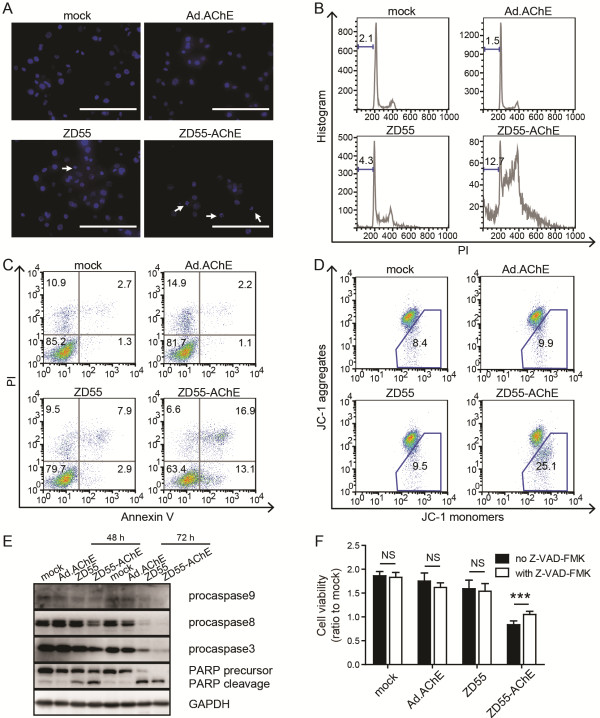
**ZD55-AChE induced gastric cancer cell apoptosis through mitochondrial pathway. (A)** Observation of the nucleic fragmentation in adenovirus-infected AGS cells by Hoechst333258 staining assay. Arrows indicated fragmented nucleic. Scale bar: 200 μm. **(B)** Measurement of the cell cycle distributions of adenovirus-infected AGS cells by PI staining. Numbers are the proportion of cells in sub-G1 phase. **(C)** Alteration of the proportion of Annexin V positive cells by adenoviruses. Numbers in the first and fourth quadrants are the proportion of apoptotic cells. **(D)** ZD55-AChE changed mitochondrial membrane potential (Δψm) of AGS cells. Numbers in the trapezoid region indicate the ratio of cells with disrupted Δψm. **(E)** Western blot of apoptosis-associated proteins. GAPDH was used as an internal control. **(F)** Caspase inhibitor Z-VAD-FMK alleviated the cytotoxicity of ZD55-AChE. The results are expressed as mean ± SD (n = 3). ***P < 0.001, NS: non-significant. AGS cells were infected with the indicated adenovirus at a MOI of 2 for 48 hours **(A**-**F)** or 72 hours **(E)**.

It was reported that AChE protein participated in apoptosis [6], and we sought to test whether ZD55-AChE induces cancer cell apoptosis. We performed Hoechst333258 staining, and found that ZD55-AChE obviously induced nucleic fragmentation in AGS cells at a MOI of 2 (Figure 4A). Moreover, the sub-G1 populations and AnnexinV positive AGS cells were increased after ZD55-AChE infection (Figure 4B, C). We next performed JC-1 staining to investigate the changes in the mitochondrial membrane potential (Δψm). The proportion of AGS cells with loss of mitochondrial membrane potential was increased in the ZD55-AChE infected cells, indicating the involvement of mitochondria in the apoptosis process (Figure 4D). ZD55-AChE reduced the procaspase 9 protein, which is the essential initiator caspase required for apoptosis signaling through the mitochondrial pathway (Figure 4E). The expression of procaspase 8, procaspase 3 and the precursor of poly ADP-ribose polymerase (PARP) were also decreased after ZD55-AChE treatment (Figure 4E). The caspase inhibitor Z-VAD-FMK alleviated the cytotoxicity of ZD55-AChE in AGS cells (Figure 4F). These data suggested that ZD55-AChE induced AGS cell apoptosis most likely through mitochondrial pathway. Notably, Ad.AChE did not induce AGS cell apoptosis at a MOI of 2 (Figure 4A-E), which is consistent with our previous data that Ad.AChE inhibited cell growth only at a high MOI level (Figure 2C, D).

### ZD55-AChE inhibited gastric cancer stem cell growth

**Figure 5:**
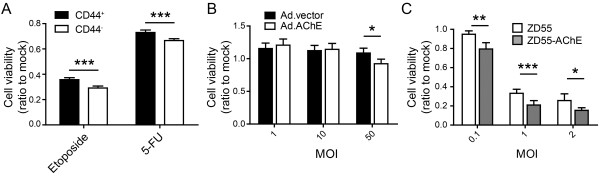
**ZD55-AChE inhibited the growth of gastric cancer stem cells. (A)** CD44^+^ AGS cells resisted to chemo-therapeutic drugs. Cells were treated with etoposide (5 μg/mL) or 5-FU (1 μg/mL) for 48 hours, and the cell viability were measured by MTT assay. **(B)** Ad.AChE inhibited CD44^+^ AGS cell growth as measured by MTT assay. **(C)** ZD55-AChE potently reduced the cell viability of CD44^+^ AGS cells as measured by MTT assay. CD44^+^ AGS cells were infected with the indicated MOI of adenovirus for 96 hours. MTT experiments were repeated 3 times. Data are shown as fold change relative to that of mock-treated cells. All data shown represent mean ± SD (n = 3). *P < 0.05, **P < 0.01, ***P < 0.001.

Increasing researches certified that the quiescent cancer stem cells are critical in cancer initiation, progression, drug resistance and relapse. Since adenovirus can infect quiescent cells, we sought to explore whether ZD55-AChE could inhibit the AGS stem cell growth. AGS cells were sorted based on the cell surface marker CD44 to obtain stem cells [14]. Both CD44^+^ and CD44^-^ cells were collected and treated with chemo-therapeutic drugs, etoposide and 5-FU. Compared to CD44^-^ cells, CD44^+^ AGS cells afforded resistance to chemo-drugs, which is one of stem cells characteristics [15] (Figure 5A). Ad.AChE decreased CD44^+^ AGS cell growth at a MOI of 50 (Figure 5B). Importantly, ZD55-AChE significantly suppressed stem cell growth at a MOI of 0.1 compared to the control virus (Figure 5C).

## Discussion

The well-known function of AChE is to hydrolyze ACh and terminate its neuronal and non-neuronal signaling transmission. Recent studies have disclosed its roles in prolonging survival time in cancer patients and promoting apoptosis [5, 16]. To our knowledge, AChE was applied to treat liver cancer delivered by nanoparticles [17, 18]. However, no cancer therapy research using viral vector to deliver AChE has been reported. In the present study, two adenoviral vector, including replication-deficient adenoviral (Ad) and oncolytic adenoviral vector (ZD55), were used to express AChE gene. ZD55-AChE presented significantly cytotoxicity to gastric cancer cells in vitro and in vivo at a low MOI level (Figure 3C-E, G), and induced mitochondrial apoptosis (Figure 4).

ACh is not only function as a neurotransmitter in the nervous system, but also involved in some critical signal pathways in cancer cells. ACh stimulates its receptors, nAChR and mAChR, and leads to the activation of the ACh receptor signaling pathway. The precise level of ACh is controlled by the AChE protein. Abnormal expression of AChE will result in the imbalance of ACh degradation and lead to the over-activation of ACh receptors [19]. Aberration of AChE activity in various types of cancers was reported. The lymphocytes from CLL patients showed significantly lower AChE activity than normal [20]. In comparsion of 55 paired tissues of healthy and cancerous gut, 32% decrease of AChE activity was observed in cancerous gut [21]. Increased ACh was found in lung cancer tissues due to the abnormal expression of AChE [22]. Lower level of AChE in cancer tissues and the reverse correlation of AChE expression with survival time were reported in liver cancer patients [5]. In our work, lower expressed AChE protein was found in gastric cancer tissues than adjacent non-cancerous tissues. Patients with high level of AChE protein showed longer survival time (Figure 1). The correlation of AChE expression and prognosis in gastric cancer patients is similar to liver cancer. Therefore, introduction of the AChE protein back may lead to the growth inhibition of cancer cells.

There are three types of AChE mRNA variants, AChE-T, AChE-R and AChE-H [23], which was able to be identified by sendimentation assay [24]. AChE-T, but not AChE-H or AChE-R, is able to anchor to the synaptic membranes or the neuromuscular junctions through ColQ or PRiMA. Only the AChE-T is regarded as truly “synaptic” [25]. Besides, AChE-R promotes cell proliferation and makes cell resistance to genotoxic stress [26, 27], however, AChE-T showed the opposite effect [28]. In our work, AChE-T was expressed.

The intracellular level of ACh were measured in various types of cells to get to know whether it related to the response of cancer cells to AChE overexpression. AGS cells expressed high level of ACh (Additional file 2: Figure S2A), and were sensitive to AChE overexpressed by viral vector, suggesting that AChE may inhibit cell growth through hydrolyzing ACh.

Studies indicated that AChE is overexpressed in apoptotic cells [6]. Absence of AChE prohibited stress-induced cells apoptosis [8]. It was also reported that overexpression of AChE renders the cells vulnerable to apoptosis, but cannot initiate apoptosis [29]. A stimulus enabling the translocation of AChE into the nuclei may be required to initiate apoptosis [8, 29]. Adenoviral vector was commonly used to express therapeutic genes in cancer therapy. On the other hand, the viral vector can provide the requisite stimulus for initiating apoptosis by itself [30]. We noticed that Ad.AChE inhibited cancer cell growth at a high MOI level (Figure 2C, D). This illustrated the stimulus effect of the viral vehicles. However, the high MOI request limited Ad.AChE to be further used in cancer therapy.

We found that ZD55-AChE induced the loss of mitochondrial membrane potential and decreased procaspase 9 and procaspase 3 proteins in AGS cells (Figure 4D, E), indicating the anti-tumor function of ZD55-AChE is associated with induction of mitochondrial apoptosis. Our data partially consistent with previous reports that AChE is required for apoptosome formation [31]. Other researches indicated that ACh stimulated the nicotinic acetylcholine receptor (nAChR) signaling which is pathologically over-activated in tumor [1]. Cholinergic signaling stimulation can promote progression in various types of cancers [32, 33]. Nicotine binds to the nAChR, activates Ca^2+^ channels and triggers the secretion of growth factors including vascular endothelial growth factor (VEGF), epidermal growth factor (EGF) and fibroblast growth factor 2 (FGF2). These autocrines powerfully trigger cell proliferation and tumor angiogenesis [34–36]. Additional work will be required to explore the anti-angiogenesis function of AChE in cancer therapy.

AChE is able to reduce intestinal cell or stem cell differentiation [37, 38], and inhibit signal transduction via PI3K/Akt pathway, which is the critical pathway in cancer stem cell maintenance [39]. Since cancer stem cells play a major role in cancer progression, metastasis and relapse [40], it is crucial to target cancer stem cells in cancer therapy. In our work, ZD55-AChE effectively inhibited cell viability of CD44^+^ AGS cells in vitro (Figure 5C). The function of AChE on cancer stem cells will be further validated in vivo.

## Conclusions

We reported here that overexpression of human AChE protein mediated by oncolytic adenovirus suppressed cancer cell growth. Oncolytic adenoviral vector ZD55 seems to be a stimulus for initiating the anti-tumor effect of AChE protein. ZD55-AChE may be an effective therapeutic method for digestive system cancers.

## Electronic supplementary material

Additional file 1: Figure S1: Ad.AChE inhibited cell growth of digestive system associated cancer cells. (**A**) Schematic diagram of the construction of Ad.AChE. Ad.WT, wild type adenovirus. Ad.vector, control adenovirus with E1 region deletion. (**B**) Verification of the inserted AChE gene by PCR assay. H_2_O was used as a water template for PCR. (**C**) Cell viability of Huh-7, SMMC-7721, SW480 and BxPc-3 cells 4 days after the indicated MOI of adenovirus infection measured by MTT assay. (**D**) Ad.AChE suppressed SW480 cell growth at a MOI of 100 as measured by MTT assay. (**E**) Detection of the cytotoxicity of Ad.AChE on normal primary fibroblast cells by MTT assay. (**F**) Morphology of normal primary fibroblast cells 4 days after adenovirus infection. Scale bar: 100 μm. MTT experiments were repeated 3 times. Data in **C**, **D, E** are shown as fold change relative to that of mock-treated cells. All data shown represent mean ± SD (n = 3). *P < 0.05, **P < 0.01, ***P < 0.001. NS: non-significant. (TIFF 3 MB)

Additional file 2: Figure S2: The intracellular level of ACh in various cell lines. (**A**) The intracellular level of ACh in gastric cancer cell lines (AGS, MGC80-3 and N87) and normal epithelia cell line (GES-1). (**B**) The intracellular level of ACh in non-gastric cancer cell lines (BxPC-3, HCT116, SMMC-7721, SW480 and Huh-7). All data shown represent mean ± SD (n = 3). *P < 0.05, ***P < 0.001. (TIFF 781 KB)

Additional file 3: Figure S3: ZD55-AChE had cytotoxicity on digestive system associated cancers. (**A**) Schematic diagram of the construction of ZD55-AChE. Ad.WT, wild type adenovirus. ZD55, control adenovirus with E1B55K region deletion. (**B**) Verification of the inserted AChE gene by PCR assay. (**C**) Cell viability of Huh-7, SMMC-7721, BxPc-3, SW480 and HCT116 cells 4 days after the indicated MOI of adenovirus infection measured by MTT assay. (**D**) Detection of the cytotoxicity of ZD55-AChE on normal primary fibroblast cells by MTT assay. MTT experiments were repeated 3 times. Data are shown as fold change relative to that of mock-treated cells. All data shown represent mean ± SD (n = 3). **P < 0.01, ***P < 0.001. NS: non-significant. (**E**) Morphology of normal primary fibroblast cells 4 days after adenovirus infection. Scale bar: 100 μm. (TIFF 3 MB)

Additional file 4: Figure S4: Representative immunohistochemistry staining images of AChE expression in xenograft tumor sections. Scale bar: 50 μm. (TIFF 3 MB)

## Abbreviations

AChE: Acetylcholinesterase
ACh: Acetylcholie
nAChR: Nicotinic acetylcholie receptor
mAChR: Muscarinic acetylcholine receptor
CT: Cancerous tissues
ANCT: The adjacent non-cancerous tissues
VEGF: Vascular endothelial growth factor
EGF: Epidermal growth factor
FGF2: Fibroblast growth factor 2
PARP: Poly ADP-ribose polymerase.
