# Supplementary figures and images for: Acetylcholinesterase overexpression mediated by oncolytic adenovirus exhibited potent anti-tumor effect
Source: BMC Cancer. 2014 Sep 15;14:668. doi: 10.1186/1471-2407-14-668 (PMC4169801; doi:10.1186/1471-2407-14-668)

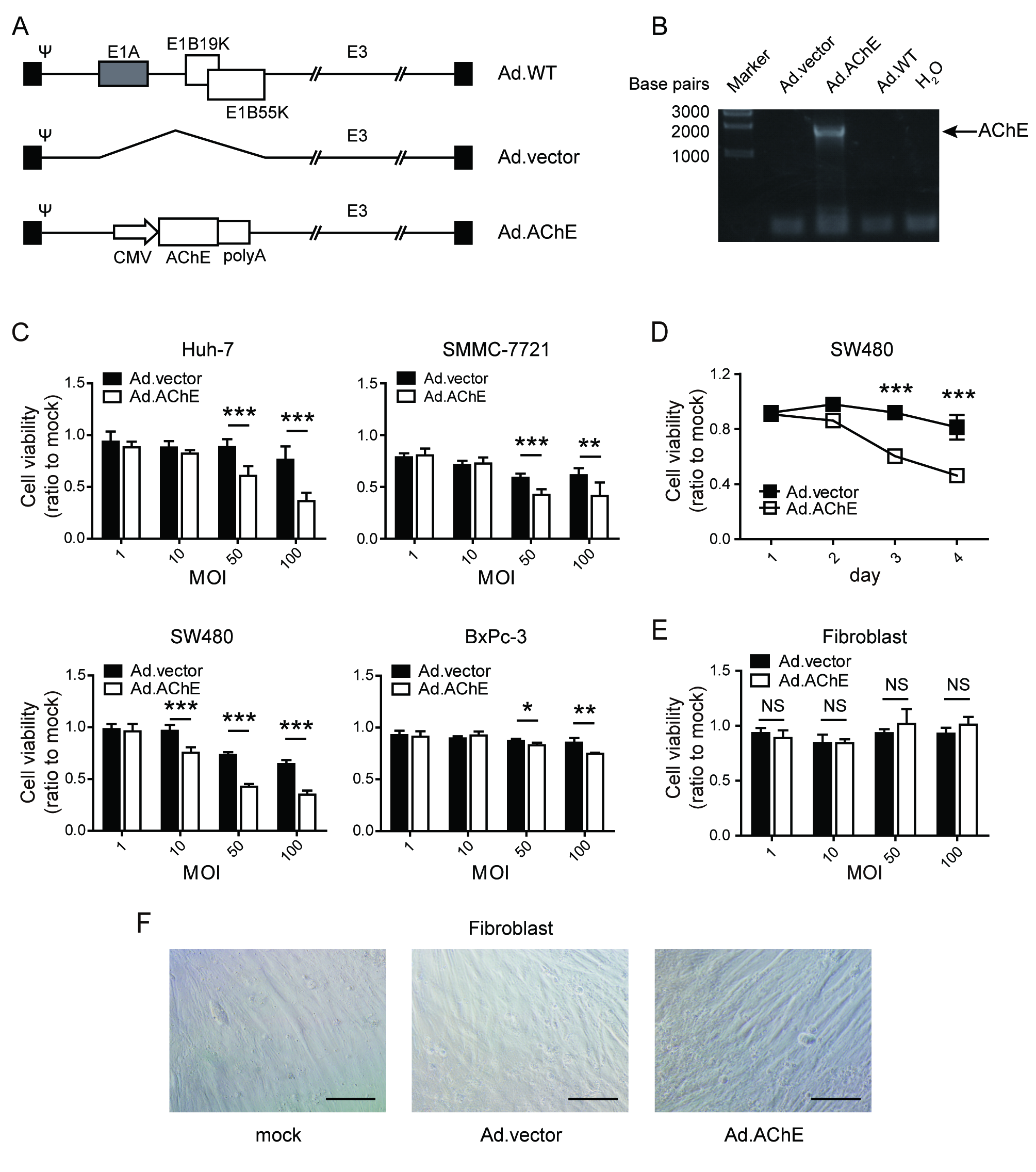

Supplement: Supplementary file 1 — Additional file 1: Figure S1: Ad.AChE inhibited cell growth of digestive system associated cancer cells. (A) Schematic diagram of the construction of Ad.AChE. Ad.WT, wild type adenovirus. Ad.vector, control adenovirus with E1 region deletion. (B) Verification of the inserted AChE gene by PCR assay. H2O was used as a water template for PCR. (C) Cell viability of Huh-7, SMMC-7721, SW480 and BxPc-3 cells 4 days after the indicated MOI of adenovirus infection measured by MTT assay. (D) Ad.AChE suppressed SW480 cell growth at a MOI of 100 as measured by MTT assay. (E) Detection of the cytotoxicity of Ad.AChE on normal primary fibroblast cells by MTT assay. (F) Morphology of normal primary fibroblast cells 4 days after adenovirus infection. Scale bar: 100 μm. MTT experiments were repeated 3 times. Data in C, D, E are shown as fold change relative to that of mock-treated cells. All data shown represent mean ± SD (n = 3). *P < 0.05, **P < 0.01, ***P < 0.001. NS: non-significant. (TIFF 3 MB) [file 12885_2014_4848_MOESM1_ESM.tiff]

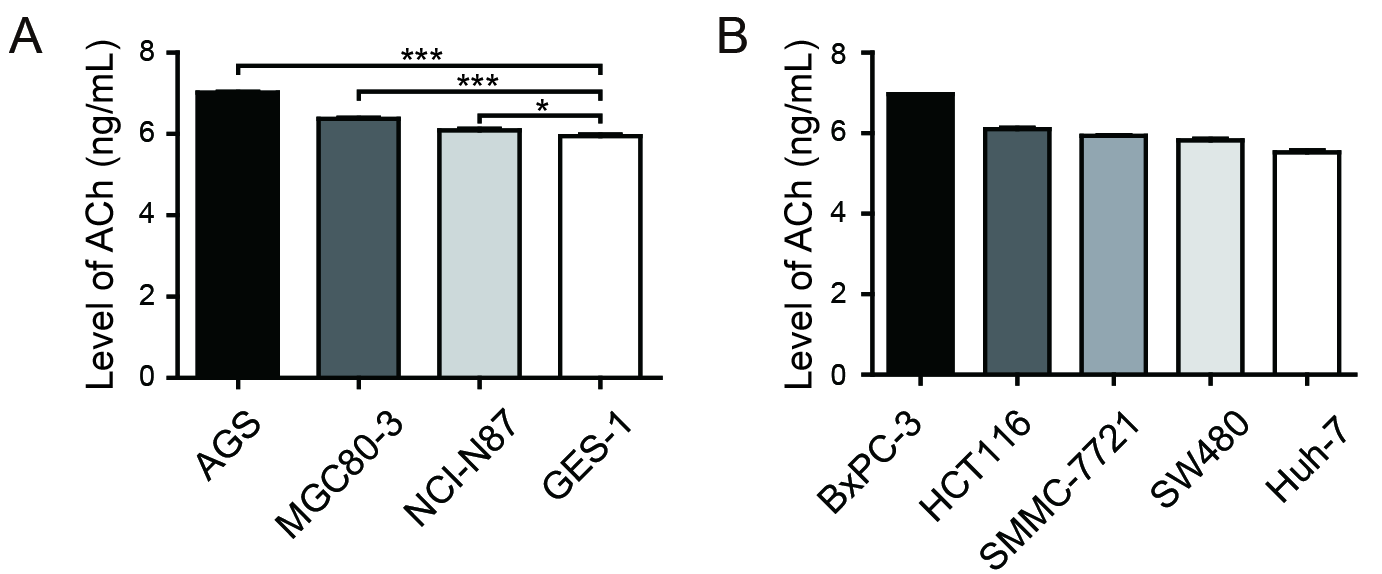

Supplement: Supplementary file 2 — Additional file 2: Figure S2: The intracellular level of ACh in various cell lines. (A) The intracellular level of ACh in gastric cancer cell lines (AGS, MGC80-3 and N87) and normal epithelia cell line (GES-1). (B) The intracellular level of ACh in non-gastric cancer cell lines (BxPC-3, HCT116, SMMC-7721, SW480 and Huh-7). All data shown represent mean ± SD (n = 3). *P < 0.05, ***P < 0.001. (TIFF 781 KB) [file 12885_2014_4848_MOESM2_ESM.tiff]

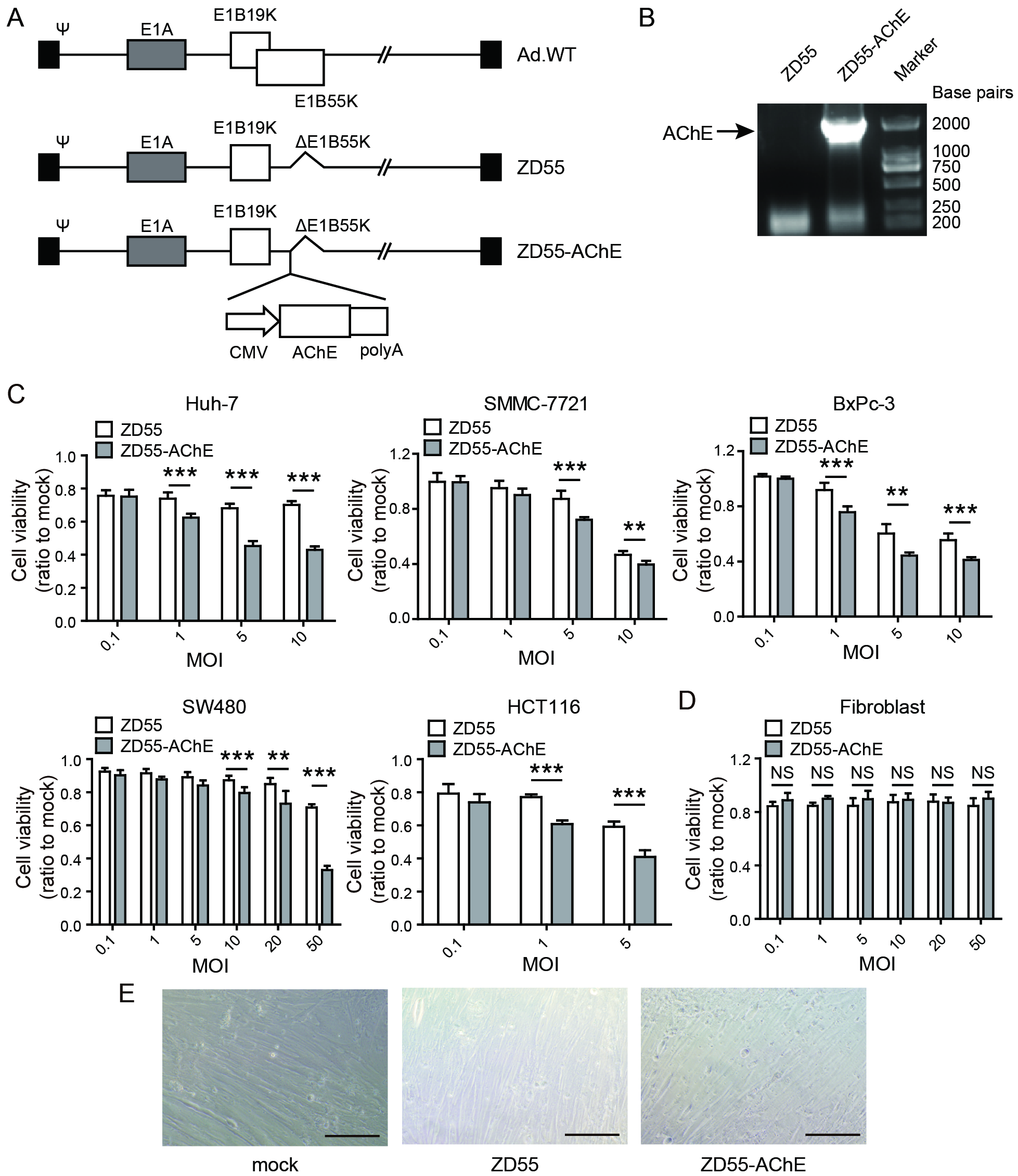

Supplement: Supplementary file 3 — Additional file 3: Figure S3: ZD55-AChE had cytotoxicity on digestive system associated cancers. (A) Schematic diagram of the construction of ZD55-AChE. Ad.WT, wild type adenovirus. ZD55, control adenovirus with E1B55K region deletion. (B) Verification of the inserted AChE gene by PCR assay. (C) Cell viability of Huh-7, SMMC-7721, BxPc-3, SW480 and HCT116 cells 4 days after the indicated MOI of adenovirus infection measured by MTT assay. (D) Detection of the cytotoxicity of ZD55-AChE on normal primary fibroblast cells by MTT assay. MTT experiments were repeated 3 times. Data are shown as fold change relative to that of mock-treated cells. All data shown represent mean ± SD (n = 3). **P < 0.01, ***P < 0.001. NS: non-significant. (E) Morphology of normal primary fibroblast cells 4 days after adenovirus infection. Scale bar: 100 μm. (TIFF 3 MB) [file 12885_2014_4848_MOESM3_ESM.tiff]

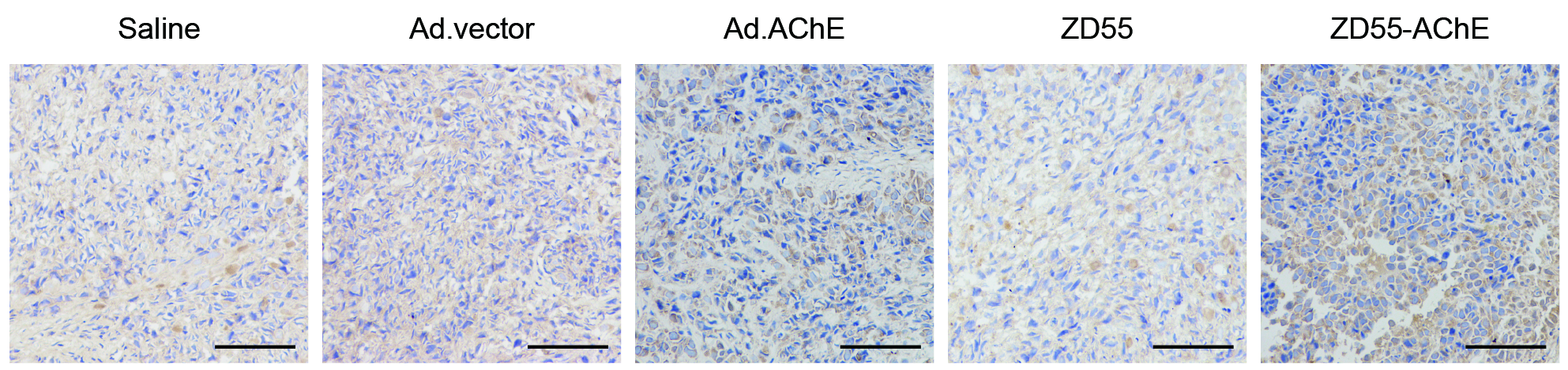

Supplement: Supplementary file 4 — Additional file 4: Figure S4: Representative immunohistochemistry staining images of AChE expression in xenograft tumor sections. Scale bar: 50 μm. (TIFF 3 MB) [file 12885_2014_4848_MOESM4_ESM.tiff]
